# Critical Commentary: Cities in a post-COVID world

**DOI:** 10.1177/00420980211018072

**Published:** 2021-06-27

**Authors:** Richard Florida, Andrés Rodríguez-Pose, Michael Storper

**Affiliations:** University of Toronto, Canada; London School of Economics, UK; London School of Economics, UK; UCLA, USA

**Keywords:** cities, COVID-19, pandemic, remote work, urban structure

## Abstract

This paper examines the effect of the COVID-19 pandemic and its related economic, fiscal, social and political fallout on cities and metropolitan regions. We assess the effect of the pandemic on urban economic geography at the intra- and inter-regional geographic scales in the context of four main forces: the social scarring instilled by the pandemic; the lockdown as a forced experiment; the need to secure the urban built environment against future risks; and changes in the urban form and system. At the macrogeographic scale, we argue the pandemic is unlikely to significantly alter the winner-take-all economic geography and spatial inequality of the global city system. At the microgeographic scale, however, we suggest that it may bring about a series of short-term and some longer-running social changes in the structure and morphology of cities, suburbs and metropolitan regions. The durability and extent of these changes will depend on the timeline and length of the pandemic.

## 摘要

本文研究新冠肺炎大流行的影响及其对城市和大都市地区造成的相关经济、财政、社会和政治后果。我们在四种主要力量的背景下，在区域内和区域间地理规模上评估大流行对城市经济地理的影响。这四种力量为：大流行造成的社会创伤；作为一种强迫实验的封锁；确保城市建筑环境免遭未来风险之害的必要性；以及城市形态和系统的变化。在宏观地理规模上，我们认为大流行不太可能显著改变赢家通吃的经济地理和全球城市体系的空间不平等。然而，在微观地理规模上，我们认为它可能会给城市、郊区和大都市地区的结构和形态带来一系列短期的、以及某些长期的社会变化。这些变化的持久性和程度将取决于大流行的时间表和长度。

## Introduction

COVID-19 is not the first virus to strike our cities, nor will it be the last. Over the course of history, cities have often been hotbeds of contagion. The Black Plagues of the 14th century killed one-third of Europe and the Middle East. The Cholera outbreaks of the 19th century decimated London, Paris, Moscow, Hamburg, New York and Chicago, among other large cities. The 1918–1920 Great Flu took the life of 50 million worldwide. In the USA it killed more people than the two World Wars and the Korean and Vietnam wars combined. It ravaged cities such as Pittsburgh, Philadelphia, Louisville and Nashville (Correia et al., 2020). Yet, the current pandemic is also leaving profound scars in many of our cities. In Bergamo, Italy, excess deaths in March 2020 were 6.67 times higher than in a normal March (McCann et al., 2020). Between March 2020 and January 2021, the USA saw 424,000 more deaths than would have occurred in a normal period, an 18% increase from baseline. New York City and New Jersey were particularly hard hit, with excess deaths 63% and 34% above normal, respectively (Katz et al., 2021). In March 2020, alone, New York City saw 3.3 times more excess deaths than resulted from the 9/11 attacks (McCann et al., 2020). Though subsequent waves or phases of the pandemic also hit smaller cities and rural areas hard.

Past pandemics wreaked havoc and influenced substantial cultural, political and urban design changes, but none of them dented the role of large cities in society. No pandemic, natural disaster or war has ever managed to stifle their growth and pre-eminence over the long term. Claims abound that ‘this time is different’, because this is the first pandemic to occur when there is a widely available alternative to face-to-face work and an alternative to doing one’s own shopping. But there is a long history of failed forecasts that ‘this time, distance is dead’; indeed, with every major improvement in transport and telecommunications capacity in the past two centuries, there has been an increase in urbanisation (Leamer and Storper, 2001). It is therefore unlikely that COVID-19, despite the high levels of devastation it has caused in certain cities, will derail the long-standing process of urbanisation and the economic role of cities. Innovation, creativity and economic growth require the clustering of talent and economic assets, face-to-face interaction, buzz, diversity and the critical mass that only cities can provide (Storper and Venables, 2004). Perhaps, paradoxically, the more efficient transportation and telecommunication technologies become in spreading out certain kinds of routine interactions, the more we invent creative new cutting-edge collaborations that demand face-to-face interaction. This is fundamentally why, throughout history, large cities have rebounded from the devastation of epidemics and many other types of crises and catastrophes.

Nonetheless, even if large cities are unlikely to lose their prominent role, they will be transformed and changed – in the short term, and even well after mass immunity. The current pandemic is producing four main forces that have the potential to lead to relatively long-lasting transformations of cities and regions as we currently know them. These four forces are:

1. Social scarring: The fear instilled by the pandemic may pull citizens apart and cause people to avoid crowded spaces for a certain post-pandemic period. This is likely to influence residence choice, travel and commute patterns, and the economic viability of certain businesses and social gathering spaces.
2. The forced experiment for employment, shopping, workplace and residence choice, and commuting, of the lockdown: Lengthy confinements have triggered a forced – or, as some will say, a ‘natural’– experiment. Workplaces and classrooms have transitioned to remote, shopping to delivery and social life has become played out largely over digital media. These changes – many of which have proven more seamless than expected – will leave a legacy on how we interact, work, shop and, consequently, live. Lockdowns have shown that there are radically different ways of living and working made possible by digital tools. However, the extent to which these alternatives will be complements or substitutes for traditional ways of interaction remains to be seen. There are signs that, for many types of work, socialisation and leisure, distanced interaction is not a full substitute and that many are eager to return to face-to-face.
3. The need to secure the urban built environment against this and future health and climate risks: Public infrastructure, public-facing businesses and all manner of spaces where lots of people gather require changes to facilitate social distancing and adequate hygiene standards. In the long-run, this crisis will prompt architects, designers and planners to consider more seriously – as was the case in previous pandemics such as the 19th-century cholera outbreak – permanent interventions that respond to the threat of future pandemics and climate risks.
4. Changes to urban built form, real estate, design and streetscapes: Physical and social distancing shape different configurations of indoor and outdoor spaces. At least some of these changes will be preserved after the immediate threat passes, whether for their public health benefits or because people simply enjoy them. The outcome of the forced experiment could also lead to more permanent changes in how and where people live and work.

This essay examines the potential immediate and long-term effects of the pandemic on cities and regions across two geographic scales. The first is concerned with the large-scale arrangement of human and economic activity across the landscape, at the inter-regional scale or what we will call the ‘macrogeographic’ scale. It seeks to understand the sorting of population, and employment and activity, into cities of different sizes and their different economic attributes. The second focuses on the arrangement of people and activity (and hence land use) within urban regions, between central and suburban areas, and at the finer granularity of neighbourhoods and districts. We examine these issues through the lens of urban and economic geography, by which we seek to contextualise limited evidence from short-term trends in a longer-term dynamic perspective. Even though the pandemic is now one year from inception at the time of this writing and is not yet over, the evidence we have is short-term and thus the best that this paper can do is a theoretically and historically informed inference about the medium-term impacts on cities after the effective end of the pandemic. The main assumption that guides our exercise is that the emergency or shut-down phase of the pandemic will end as vaccination programmes reach the majority of the population. Indeed, as we finalise this paper mass vaccination is proceeding in many countries. Our primary focus is on the advanced nations of Western Europe and North America. Many of the issues we discuss apply more widely, but in parts of the world with very different levels of income, economic development, state capacity, and rules and institutions, not to mention different starting points in terms of urban infrastructure, housing and overall quality of built environments, our inferences could require substantial modification.

Based on these assumptions, we take our best shot at more and less likely scenarios as the four forces play out at two scales, and reach some conclusions about post-COVID geography. Though there is considerable uncertainty attached to this type of analysis, we believe that knowledge of the fundamentals of urbanisation and the limited short-term evidence can generate educated guesses. Timely analysis is necessary. Past experience of urban and economic shocks shows that the way public policy reacts to acute shocks may actually shape the long term, in much the same way that counter-cyclical economic policy may prevent transitory crises from becoming long-term depressions. We will argue that there are potential unnecessary losses in urban efficiency and quality of life that can be avoided with good short-term policy. It would be unwise to allow unnecessary and avoidable urban crises of the past to repeat themselves in a post-COVID future.

## Pandemic geography: What we know so far

SARS-CoV-2 is the deadliest viral pandemic since the emergence of HIV in 1981 and the worst airborne virus since the Great Flu of 1918–1920. It is the first time an airborne pandemic has gone global in the age of widespread commercial air travel (Rosenwald, 2020).

Big cities, with a very high degree of air connectivity, many international travellers and people in close proximity, were hit hardest during the first wave of the pandemic in the Western world in March and April 2020. There is a great difference between the geography of the first-hit places and the ultimate geography of infections. For the first-hit places, the severity of the outbreak appears less a result of density and more a result of their greater connectivity to the world and highly interactive creative local economies. Nonetheless, early analyses argued that the density of first-hit places was a fundamental driver of infection. Nathan (2020) contends, correctly in our view, that this is an ecological fallacy. There has been a weakening relationship between density and infection rates over time. In subsequent waves, many small cities and rural regions were hard-hit in per capita terms. The Dakotas, two of the most isolated and sparsely populated states in the USA, became the epicentre of the autumn surge. The severity of the outbreak there was not attributed to density but to policies such as the lack of state-wide mask mandates (Bruni, 2020). In the first national COVID-19 study developed in Spain, Soria (population density 9.2 hab./km^2^) was the hardest-hit province, with the share of its population with antibodies after exposure to the virus standing at almost three times the Spanish average (Ministerio de Sanidad, 2020). East Asian cities in general, including Hong Kong, Singapore and Tokyo, are a testament to the fact that density does not equal destiny during this pandemic (Patino, 2020a).

Aside from a swift, decisive response from local and national leaders and strong adherence to public health policies, a few specific factors stand out as potential determinants of which cities – and which communities within cities – experience the most severe outbreaks.

The first is connectivity. Cities that became early epicentres are global hubs for business and tourism, with some of the world’s busiest airports. Research indicates that – as happened earlier in the case of the SARS epidemic (Harris Ali and Keil, 2006) – it was this type of global connectivity which led to the initial outbreaks in large cities such as New York and London (Nathan and Overman, 2020). Similarly, many smaller communities that experienced severe early outbreaks were also globally connected, for example ski resorts in the Austrian Alps and US Rockies, which attract affluent skiers and played host to early ‘super spreader’ events (Branch, 2020; Hoffower, 2020). These places were hit before preventive strategies and better treatment became available. Less connected places generally were hit later, when control measures (of different levels) were already in place.

The type of density may matter more than the level. First is the issue of work density versus residential density. The combination of connectivity and type of work, at a time when distancing measures were not in place, made large cities ideal targets for severe outbreaks (Glaeser, 2020; Hamidi et al., 2020).

The type of prevalent work has also shaped the incidence of the pandemic. As office-based workers increasingly stayed at home and were able to work remotely, public-facing workers remained most exposed to the virus at work. Often, little was done to limit interactions in risky high-contact spaces between these workers and the public, or among the workers. And while many of these spaces bring people of different social classes into contact, the impact of the virus has diverged according to geography and social class, with the least privileged people and places normally seeing the worst effects. One is more likely to catch the virus working all day in a restaurant than while going there briefly as a customer. White collar knowledge workers are more likely to be able to work from home, to have access to a personal car for transportation and to live in uncrowded homes. However, the differential impact of the spread of the virus is also conditional on availability and access to health services in different parts of a city and by different income groups.

**Figure 1.**
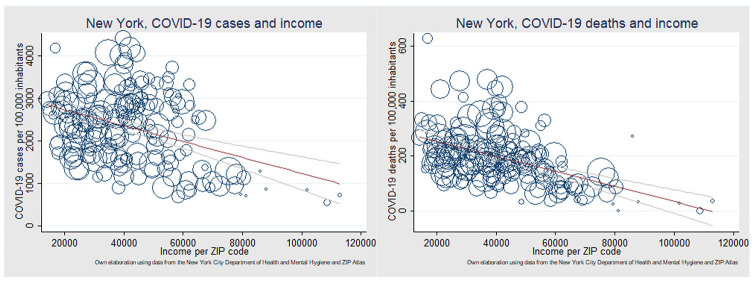
Neighbourhood wealth and incidence of COVID-19 in New York City during the first wave of the pandemic. Situation as of 13 June 2020

Thus, from the beginning, in many of the hardest-hit places, the urban geography of infection became highly stratified by socio-economic group and location or neighbourhood. This was, for example, the case of New York City, where the ZIP codes with the lowest income per capita saw the biggest incidence of both COVID-19-related cases and deaths in the early stages of the pandemic (Figure 1). Poorly paid service workers are preyed upon by the virus on their often-lengthy public transit commutes and their essential, in-person jobs – stocking supermarkets, clerking pharmacies, cleaning, collecting rubbish, driving buses and trains and, last but not least, performing all types of jobs in hospitals. Immigrants are disproportionately represented in these fields of work.

The high-risk settings to which low-wage workers are routinely exposed are compounded in many cases by high-risk home environments, with many people living in overcrowded conditions where multiple members of the household routinely leave the home for work. Thus, residential density may not play a big role in infection but residential crowding does.

**Figure 2.**
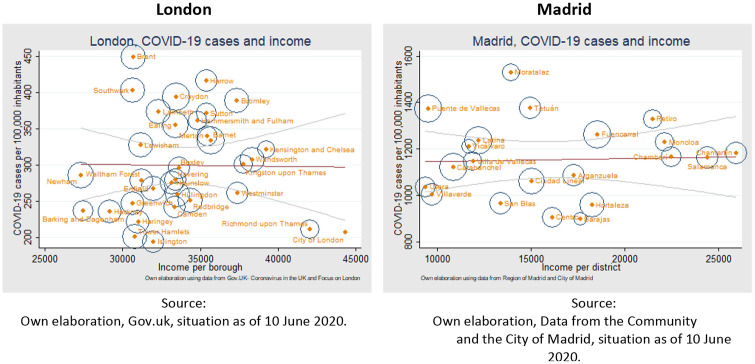
COVID-19 cases per 100,000 inhabitants by neighbourhood in London and Madrid and income per capita in the first wave of the pandemic.

The wealth division is also shaped in part by local context. In some of the worst hit European cities the inequality dimension in terms of incidence of coronavirus, while present, was far less marked than in New York City during the first wave. In two of the worst hit European cities, Madrid and London, although working-class neighbourhoods with large proportions of essential service workers and large shares of overcrowded housing were stricken, so were many better-off areas of the city (Figure 2). Universal access to healthcare, which encouraged individuals presenting symptoms to go to hospital in these two cities, in contrast to the uncertainty looming over many uninsured workers in New York, may have played a role in protecting those at the bottom of the income scale more.

Community-based interactions have also counted in infection and death. Ding et al. (2020) demonstrate that tightly linked communities with high levels of social capital, expressed through frequent face-to-face interactions of family, ethnic group or religious groups, have high levels of transmission. High sociability has been also connected to excess mortality in the case of Europe (Rodríguez-Pose and Burlina, 2021). This is paradoxical, in that in many other areas of life, such strong communities have been shown to be beneficial for physical and mental health and economic achievement (Putnam, 2000). In the case of a pandemic, frequent meetings and large family gatherings make them more vulnerable than more atomised populations or nuclear families.

Thus, the pure urban physical structure – as measured by density or transit mode – may contribute somewhat to the pandemic but it is the structure of interactions – from global to regional to neighbourhood to family – that determine its spread, and underlying socio-economic conditions and public policies that shape its severity once spread. Socio-economic conditions and policies have strong geographical variation, as do underlying structures of interaction.

## Exploring the potential consequences of the pandemicfor cities and regions

We now turn to the consequences of the pandemic for the way we live and work and, therefore, for the structure and configuration of our cities. This is not an exhaustive catalogue of changes to cities but reflects behaviours and practices that can reshape urban geography. We deal with these at two scales of outcome: the macro- (inter-regional) and micro- (neighbourhood, district) urban geography, where the unifying theme is how the spatial arrangement of households, work, leisure and interactions may change and, with it, the economic geography of areas within and between metropolitan areas. We look beyond the initial emergency phase of the pandemic, involving strong restrictions on daily life and interactions that occurred roughly from early 2020 to mid-2021. And toward the more enduring and durable geographic changes that may emerge as the world shifts back to greater mobility, connectivity and interaction by late 2021 or early 2022.

### Social scarring

Social scarring refers to the fears that humans – and, especially, residents of large cities – have acquired during this pandemic, the most pronounced of which is ‘enochlophobia’, or fear of crowds. Social scarring is likely to affect people as long as the risk of infection remains and, for some, for a considerable time afterwards.

The majority of the population is likely to bounce back quickly, especially in cities, as has been the case in previous pandemics. Cities provide a space for humans to interact, to feel they are alive, to mingle, laugh and love. Of course, for those who are victims of severe poverty and institutionalised racism, these qualities of cities are also combined with violence of many types. For all populations, these basic human needs have been in short supply during confinement, especially in societies in which the number of people living alone continues to increase. Social scarring will, sooner or later, give way to the human need for togetherness and in-person relationships. That being said, it is possible certain scars will last lifetimes, prompting anxiety about massive crowds in places such as football stadiums, basketball arenas, rock concerts, music festivals and West End playhouses, and an increased awareness of hygiene. There may be strong cohort effects in these fears.

#### Microgeographic impacts of social scarring

During the early phases of the pandemic it is logical that city dwellers tend to look for more personal space and more private amenities, which can be found outside of cities. That, too, has happened in the wake of previous pandemics. At the beginning of this pandemic, there were reports of some ‘urban exodus’ and there was rampant speculation that this would endure. For example, The *New York Times* reported increases in numbers of households moving from New York City to suburbs in March and April 2020 (Hughes, 2020). A Harris Poll found that COVID-19 prompted nearly 40% of urban dwellers to consider moving to less crowded places. If remote work remains the norm, many of these out-movers may not return (Hart, 2020). And yet, according to data from Zillow and Apartment List from the summer of 2020, the movement away from American cities is far from widespread. Other data have indicated that the two most dense, most expensive US cities, San Francisco and Manhattan, are in a class of their own in terms of outmigration and declines in rent (Patino, 2020b). The increase in suburban property values and in the for-sale residential real-estate market writ large may have been influenced by structural economic factors, such as historically low interest rates, while falling big city rents – following so many years of gains – might indicate a broader market correction. But more recent data suggest that the urban exodus was overblown – made up largely of a temporary exodus of young people and students, and accelerated family formation moves. Data as of April 2021 suggest some movement of population back to large cities. New York City, for example, saw a strong rebound in residential sales and rentals in early 2021. In Europe, during the opening up in summer 2020 (aside from the summer holidays), crowds surged back as soon as mobility restrictions were lifted. Unfortunately, this led to a resurgence of infection.

However, over the longer term while residual fear of additional waves or of an endemic virus exists, it remains possible that a subset of urbanites may be too scared to come back to cities, while others may choose to live outside of city centres and telework almost full time. Others will no longer be able to support two homes and will give up urban apartments. The fear of being in enclosed, small apartments may outweigh the advantages of inner-city living. Similarly, the fear of enclosed subways, trains and buses may exert even more centripetal pressure, and may linger for some time. Public transit ridership remains down in major cities as of April 2022. The longer the pandemic or fear of the virus lasts, the greater this effect will be, as older, more settled populations seek safety and security over immediate urban buzz.

For younger, highly skilled workers, the calculus is likely to be different. Some households, particularly those with young children, may decamp to the suburbs or exurbs of major cities, retaining access to creative class (semi-remote) work but recalibrating access to amenities. Many suburban areas will increase their urban character and develop a variety of functional mixes (Boussauw et al., 2011). Superstar city regions, such as New York, San Francisco, Boston, London or Paris, already offer abundant suburban and exurban peripheries that could attract some highly skilled creative class members. Younger workers have also returned to cities in the wake of previous pandemics. The quality of life in pastoral suburbs may not offer immediate street life but it certainly offers a good combination of access to work, to other creatives and to the occasional inner-city excursion for culture or recreation. Access to the social and work networks of these cities would be maintained but with greater perceived safety.

But even if this scenario characterises a certain segment of the young, highly skilled workforce, it likely will not signify a generational shift on the scale of white flight in the post-war 20th century or the urban renaissance of the 21st century. There are already press accounts of this upper-middle class scrambling to find all manner of new service inputs, notably private or semi-private tutors and caregivers, and entertainment providers. The pools of such relatively skilled service workers and the variety of services they can provide are greater in the prosperous large metropolitan areas or ‘metros’. While being in a smaller metro with a bigger house may appeal on one level, access to this talent pool is less for this ambitious population. This is a type of amenity that has not yet received sustained consideration in research.

Many young, educated people may continue to move to cities and urban areas, in a process described as *youthification* (Moos, 2016). Recent research (Cortright, 2020) finds that young people with college degrees aged 25–34 years have accounted for 50% of the population increase in central urban neighbourhoods in the USA since 2010 (Baum-Snow and Hartley, 2020). These trends will likely continue. Young people are drawn to cities for the combination of economic opportunity, dense labour markets, dense mating and friendship markets, and the related amenities they provide. They will continue to come to cities after the end of the pandemic. Ahlfeldt et al. (2020) argue that *even if* employment remains permanently less dependent on face-to-face interaction, the amenity and interaction value of the city alone will keep this population in amenity-rich cities and their central areas.

#### Macrogeographic impacts of social scarring

Let us now consider a more profound alteration of the urban system, one where a high proportion of the highly educated workforce reverses the trend toward concentration in those regions and lives in other types of city-regions. In order to think this through, let us recap the fundamentals of economic geography since the 1980s.

Since 1980, a distinctive macrogeography has been generated around the world. Cities have generally prospered at the expense of rural areas. But certain cities have prospered much more than others. These are cities that generate, attract and retain the highly educated workers of the creative class (Florida, 2004). In the USA, there has been considerable turbulence in the ranks of high-income cities in the post-1980 period. Cities that were stars of the mid-20th century have fallen well down the rankings (e.g. Detroit and St Louis); others have risen (Houston); and still other formerly prosperous ones have persisted by regenerating their economies (New York, San Francisco). Overall, the inequality of incomes between superstar regions on one hand, and other cities and rural areas on the other, is at its highest in a century, and inter-regional migration at its lowest (Kemeny and Storper, 2020). Meanwhile, the geographical variance of wages for the highly educated is at a peak, meaning that for them it pays to migrate to certain cities and leave other regions behind. This is not the case for the less skilled, where wages in big cities are only marginally higher than in other, cheaper places (Autor, 2020).

Behind these striking patterns of the geography of prosperity is the geography of the Third Industrial Revolution. The technology-based, creative-class activities of this period are strongly concentrated in a necklace of superstar city-regions, generating the labour and income geography noted above.

Might social scarring undo these fundamentals of economic geography? For the advantages of these cities to decline in favour of those of other regions – smaller, less educated, less technology-intensive – two scenarios might be in play. First, highly educated and skilled workers may be so affected by a fear of density and crowds that they would prioritise safety and distance at the expense of access to higher paid economic opportunities. This may tilt the balance in favour of cities with lesser concentrations of highly paid work and networking advantages. An alternative scenario is that this trade-off between work opportunity and type of city will be voided by the large-scale substitution of telework for interactive work, which we discuss below.

### Lockdown as a forced experiment in work and commerce: A new geography of jobs?

The lockdown has become a massive forced experiment in teleworking, remote shopping, dependency on home deliveries, and even in keeping and developing personal relationships. All forms of work and commerce have been affected but, for heuristic purposes, we identify three different kinds of work in the COVID-19 era.

1. Essential work that cannot be done remotely but is not high-touch or heavily public-facing, including infrastructure, construction and maintenance.
2. High-touch, public-facing work, providing essential and non-essential services, including grocery clerks and healthcare workers in the former group, and waiters and shop clerks in the latter.
3. Knowledge work that can be done remotely or partly remotely: the highly skilled creative class.

The first of these categories saw relatively little change in work or its location, although there were new health and safety measures on the job.

In the second category, essential workplaces, including healthcare facilities, grocery stores, auto and bike repair shops, agriculture and food processing facilities, manufacturers of essential products, logistics and delivery workers, etc., needed to continue their labour, although facing increasing competition – at least in the case of grocery stores, most types of manufacturing and even healthcare facilities – from online providers. And they did so while proactively responding to the health emergency and implementing safety precautions. Logistics and delivery workers, led by companies such as Amazon, saw rapid growth.

By contrast, non-essential workplaces in the second category, including restaurants, hair and nail parlours, clothing stores, concert halls, bars, sports venues, etc., suffered large declines in demand. At this time, we cannot know how long the depression in these activities will last, and in what form high-touch urban activities generating public-facing work will return. The basic economics of these activities – involving distancing, bigger spaces and new procedures – may alter the cost structure of providing them enough to induce demand-elastic responses.

One of the challenges of the pandemic’s effect on such high-touch, non-essential work is a potential increase in already-high levels of wage and income inequality. The most at-risk, high-touch workers are at the bottom of the wage scale. They are also at the greatest risk from automation. The massive move to online shopping is accelerating the transformation of cashiers and shopping assistants into deliverers.

This has and is likely to continue to have exacerbated inequalities between an affluent and highly educated elite that can mostly telework and an army of service workers, whose duties are increasingly performed through deliveries. Mid-level high-street small-business managers will have fewer opportunities. And, whereas widening economic inequality previously affected large cities the most, now these changes will permeate small cities, towns and rural areas as well. The middle class will continue to shrink.

The third occupational category is highly educated, knowledge workers, whose work consists mostly of abstract and cognitive tasks. During the pandemic many of them have shifted to telework. Estimates are that remote work will increase from about 10% of the workforce in the USA prior to the pandemic to roughly 20% of the workforce post-pandemic with, say, another 20% or more of the workforce working remotely a few days a week. Can this create a new geography of labour, altering the fundamentals of economic geography and urban systems since 1980? This is probably the key aspect of the pandemic shock because it affects the overall economic structure and economic geography of work, concomitant residential choice behaviours, and the future of superstar metros and the urban system. It is also the subject of considerable speculation, involving the usual hyperbole of techno-optimists that ‘everything will change’, or that it will be better because of the latest technological revolution. Let us remember that the ‘death of distance’ hypothesis from the late 1980s turned out to be false, and that the optimistic predictions that the advent of the internet would generate a more informed and democratically transparent society have also turned out to be false.

During the lockdown people have been forced to live and work using technologies they were previously unfamiliar with or unwilling or reluctant to use on a regular basis. Telecommunication platforms such as Zoom or Teams have come into their own during this pandemic and have showcased a new way of interacting, living and working.

It is possible to think of a range of transformations, running from a dramatic and permanent switch to all-online in certain lines of work to a temporary online shift followed by a rebound to the pre-existing status quo. The most likely scenario is a ‘new normal’ consisting of work that has a greater mix of remote-from-home than prior to the forced experiment.

If telework proves not to adversely affect productivity and work experience, the implications for office design, commercial real estate and commuting could be considerable. Its long-term impact will depend on how companies and workers navigate the complex advantages and disadvantages of this new landscape.

For workers, distance working may increase wellbeing by reducing commuting and providing more family time. Companies will make savings from leasing less space and the efficiencies of employees using less time in meetings and chit-chat by the coffee machine or the water cooler.

On the other hand, workers might find it difficult to remain focused and productive during long periods out of the office, with employers struggling to monitor employee productivity. Additionally, face-to-face contact retains significant advantages over digital contact. Communication is more seamless using facial expressions, body language and physical presence. Face-to-face contact builds social networks, social capital and reliable trust-based relationships that facilitate the complex projects needed in creative work. Workers and students are also reporting difficulty in concentrating and less motivation, much of which comes from the co-presence, membership and identity physical workplaces offer (Valet, 2020). One year into the pandemic, there is widespread use of the term ‘Zoom fatigue’.

The jury is still out on how the pandemic’s shock to knowledge work will affect that sector in the long run. On the basis of what we know (at the time of writing), three different dimensions of knowledge-based work can be contemplated. First, consider projects already underway with colleagues that one already knows. Telework platforms are a reasonably good substitute for one-on-one or small group meetings. Here, telework complements the face-to-face parts of one’s job. In some professions, such as individual psychotherapy, the substitution effect seems close to complete, while in project-oriented work – whether within organisations or freelance – it is partial. Psychotherapists also do not yet know whether they can successfully start therapy with patients they have never met.

Second, for large group, high-contact, knowledge-based work, such as teaching, entertaining, networking, holding conventions, remoteness is a poor substitute for presence, because individual relationships are weaker and in-presence performance offers a wider band of communication than online (Storper and Venables, 2004). In teaching, moreover, it is one thing to switch to remote when student and instructor have already established relationships, but another when they expect never to meet in person.

Third, consider the incorporation of new colleagues, organising new projects and collaborations: telework is weakest here. There is little evidence that telework can successfully establish networks, socialise new participants and permit the evaluation of partners in creative work that depends on high levels of tacit knowledge and partner evaluation ‘between the lines’. Thus, some substitution of telework for direct work in all three of these dimensions will happen but in different proportions. The use of telework will certainly accelerate with the forced experiment of the pandemic. But the substitution effect will reach its limits in the second and third dimensions as time goes on, as existing activities wear down their stock of acquired networks and as the need to move on to new projects with new collaborators increases. There is also some anecdotal evidence that large tech companies have held back on recruiting new workers until they are able to reopen their offices and bring these workers into physical spaces with colleagues. Based on both the successes of having shifted to remote interaction and the mounting evidence of the limitations of such socially distanced work, new forms of hybrid work are the likely permanent outcome of the shock. Hybrid work involves working weeks that mix presential and office-based interactions and distance interactions from home or far away. Standard practice will include a remote link into meetings so that those in another locale can join, increasing meeting efficiency and the feasibility of schedules. One can imagine an intensification of work, as we will now be expected to attend office meetings even when on business trips.

#### Microgeographic consequences of the forced lockdown experiment

Even a partial shift to remote work will have a significant impact on mobility, transport and real estate. Those who can work remotely at least partially will tend to avoid crowded public transport, especially at peak hours. Likewise, those with long drives to work will be able to avoid those drives more often and travel at off-peak hours at other times. The convenience premium enjoyed by close-in city dwellers who have short, easy commutes will evaporate for those working always, or mostly, from home. Some workers might tolerate even longer commutes from even more distant locations if they only have to go in to work once or twice a week.

Fewer people coming to the office less often would depress demand for office space, especially in expensive downtown locations.

In the residential real-estate market, the highly educated and affluent populations that have been re-urbanising since the 1980s may start to see the benefits of living outside but close to major cities, fleeing gentrified neighbourhoods for upscale suburbs and nearby small towns. A large number of professionals are learning that remote work enables them to work effectively and, in many cases, increase their wellbeing by doing it from the comfort of their houses in nearby suburbs and towns. This might encourage an exodus to gentrified affluent suburbs and small towns near superstar cities – such as the Hudson Valley, Oxfordshire, Surrey and Normandy – where easy access to a superstar city is guaranteed. This would have a knock-on effect on residential house prices in city centres. The degree of this effect will greatly depend on the attitude of young professionals to inner-city living when being in the office every day is no longer necessary and/or required. If many of them leave, then real-estate prices could be significantly affected, though as we noted previously, it is still early for this effect to manifest itself in the data.

However, the long-term prospect of remote work is not the only factor many young professionals consider. Whether and to what degree urban amenities such as restaurants, bars, clubs, museums and concert venues reopen will also have a bearing on urban outmigration (Ahlfeldt et al., 2020). For some, the need to avoid public transit could inspire them to live even closer to the urban core, in neighbourhoods where they can rely exclusively on walking and biking for transportation, paradoxically generating the ‘live–work neighbourhoods’ that have been normatively promoted by urban planners for decades.

#### Macrogeographic impacts of the forced lockdown experiment

However, the geography of where remote work can take place is highly uneven. The majority of work tasks capable of being transformed relatively seamlessly into tele-jobs are in well-compensated knowledge and professional fields, concentrated in tech hubs such as San Francisco, Washington DC and Austin (Dingel and Neiman, 2020). This has been confirmed by the OECD (2020) in an analysis of the regional capacity for remote working in OECD countries. The study found that in 25 out of the 28 countries considered, capital city-regions had the highest potential for remote working. The three exceptions were Switzerland, with Zurich having the highest potential; Germany, with Hamburg; and Turkey, with Istanbul. The top region in terms of remote working capacity is Greater London, with more than 54% of all jobs in the city, with three other areas – the outskirts of Brussels, *Grand-Paris* and Stockholm – exceeding the 50% threshold (OECD, 2020) (Table 1). In California, the San Francisco region has a 50% remote work potential, while Los Angeles only a 30% level. Some of this is due to differences in high-/low-skill work; but some of it because the Los Angeles economy is more oriented toward the second dimension we explore above, that is, performance-oriented, public-facing, creative work, as opposed to office-based high-skill work.

**Table 1.** Top 15 areas by capacity for remote working in Europe and the USA.

Country	Region	Share (%)
GBR	Greater London	54.21
BEL	Walloon Brabant	50.93
FRA	Île-de-France	50.90
SWE	Stockholm	50.65
CZE	Prague	49.69
FIN	Helsinki-Uusimaa	48.82
LUX	Luxembourg	48.57
NOR	Oslo and Akershus	48.25
HUN	Budapest	47.87
CHE	Zurich	47.64
GBR	South-East England	47.32
DNK	Capital City Region	46.91
USA	District of Columbia	46.65
LTU	Vilnius Region	45.05
BEL	Flemish Brabant	44.75

*Source*: OECD (2020).

By contrast, in most Turkish and Spanish regions less than one-quarter of the workforce could easily move to teleworking during the pandemic. The same applies to the state of Mississippi in the USA, Basilicata in Italy, or West Romania (OECD, 2020).

Large cities will initially and partially suffer from these trends; San Francisco’s downtown office towers and hotels are almost empty at the time of writing, and there are widespread anecdotes of tech workers leaving for Austin and Miami during the pandemic. But the mixed models of in-person/remote working that will come to apply to all three dimensions of creative work will mean that city-centre offices will still attract high earners. In addition, in the medium-term, while a certain cohort will have discovered that life in the high-cost, fast-lane of the San Franciscos of this world is not for them, a post-pandemic cohort is likely to flood back in, as activity expands and rents are significantly lower than in pre-pandemic times. There is simply no replacing the advantages of agglomeration for establishing oneself in a knowledge or creative career and acquiring the reputation and networks to flourish there. Cultural and tourist hubs of high value, such as Florence, Venice and Barcelona, will also recover because of their sheer uniqueness and their place on so many ‘bucket lists’. Some firms will be more suitable for a longer-term transition to telework, including internet companies such as Twitter, which recently announced some employees would be permitted to work from home ‘forever’ (Kantrowitz, 2020). Other categories, by contrast, will still benefit from frequent face-to-face interaction.

The impact outside large cities will also vary. Some intermediate cities, including smaller tech hubs and university towns, will become more appealing thanks to their cosmopolitan cultures and their lack of crowding. Some individuals may choose to follow the template laid by the Tulsa Remote programme, which attempts to lure remote knowledge workers to Oklahoma city with its low cost of living (Holder, 2020). The pandemic may kick-start greater creative or knowledge-based activity in metro regions just below the superstar mega-regions as, for example, in Miami, but this is likely to be complementary to more superstar city growth, rather than a substitute for it.

Outside of these limited cases, most intermediate cities, towns and rural areas are less likely to benefit much from the advantages of remote work. First, because only a limited share of their existing workforce can telework. Second, because they lack the agglomeration economies that knowledge-intensive and creative industries require. Most large knowledge-economy firms will still require occasional face-to-face meetings and value the social networks and cultural aspects of a workforce that knows one another in person. Time zones are also a consideration. More collaborative occupational types, including executives, marketing professionals and designers, are less likely to fully embrace remote work. More technical workers, in fields such as data, software engineering and customer support, might be better candidates.

On the whole, in contrast to the micro-geographical scale, where suburbs may become more attractive to some fraction of the workforce, notably families with children, at the macrogeographical scale few declining and left-behind places will benefit from this. The economic downturn and the acceleration of automation and artificial intelligence prompted by distancing could exacerbate existing vulnerabilities of declining places. This could add urgency to the ongoing debate about the need for strategic revitalisation plans, or even greater public-sector assistance for these places.

Our main prediction is that knowledge work is likely to become more hybrid – present and distanced – with a limited trickle-down effect at the inter-metro scale as cohort effects induce some knowledge workers and entrepreneurs to seek somewhat cheaper metros, and a possible acceleration of suburbanisation of existing knowledge-based workers in superstar metros. All in all, we foresee a continuation of the macro-geographical trends of the past 40 years, and this is a warning to those forecasting the revival of regions left behind because of the pandemic (Rodríguez-Pose, 2018). The problem of severe inter-regional inequality will remain (Florida, 2017). The divides between prosperous cities and regions and struggling areas will continue and possibly even widen (Florida, 2019). More paradoxically, a ‘moving into the centre’ dynamics may emerge, as rents fall, and the value of not commuting far or on transit rises, for a certain cohort. The scarring of not having access to urban amenities may contribute to another wave of centre-city living. Finally, it should be remembered that the USA is in many ways an outlier in the world (along with Australia, Canada and New Zealand) as a much more suburban, less dense type of city life is more common there (OECD, 2020). Suburbanisation is not new but is rather the basic benchmark for these areas. The pandemic did not generate it.

### Securing the urban environment from the health shock of coronavirus: Impacts on consumption, leisure and mobility

The way we relate to each other, the way we work and the way we shop were all transformed in early 2020 in order to limit contagion. These measures not only require greater social distancing but also wellness checks and tests (often at the expense of individual liberties) and physical changes to the built environment. Most of the impacts in this area will be microgeographic by nature.

One outcome of the pandemic has been experimentation with using more of the urban space for outdoor interaction. Restaurants and stores can serve more people if they are permitted to do business in the street. Some cities pioneered this approach, many others followed suit. The Lithuanian capital, Vilnius (Henley, 2020), and Santa Barbara, California, closed downtown streets and turned them over to restaurants early on. Subsequently, all over the world, there was a boom in outdoor dining, though this ultimately gave way to shutdowns in the third wave of the virus. Still, the collateral pleasure of these extended spaces may become a permanent feature of urban landscapes everywhere. Nevertheless, stores, restaurants, theatres, museums and other gathering spaces may need to be redesigned to promote social distancing as a bulwark against future pandemics.

#### Microgeographic impacts of the health shock

The microgeographic impacts of the pandemic may be shaped by the acceleration of pre-existing shopping trends. Online shopping is becoming more common, less expensive and less arduous than shopping in person. Many restaurants, stores and cultural facilities will not survive the increased costs of doing business combined with reduced customer demand as people increasingly choose to use food delivery and home entertainment services. These developments would, in turn, bolster gig economy delivery companies, such as Uber, and digital entertainment platforms, such as Netflix.

All of this disruption and, for many, economic devastation, will create a handful of new opportunities. There will be new demand for technological and design innovations that monitor people’s movement and health and encourage people to socially distance. There will be new employment opportunities for people to implement and maintain these innovations, especially people carrying out health checks and monitoring hygiene.

The need to implement social distancing and physical barriers in public spaces such as restaurants and offices is likely to be temporary. New technologies will emerge that could have knock-on effects by helping minimise future seasonal flu epidemics and other public health concerns. Urban design and architectural standards that promote distancing and limit shared touchpoints may become permanent if people appreciate their secondary benefits – particularly wider sidewalks and safer bike lanes. Underlying these transformations is the age-old question of their social and geographical distribution. Will they occur in an unequal way, reproducing existing hierarchies of class and race, or will they create a window of opportunity for urban transformations that are more socially just?

#### Macrogeographic impacts of the health shock

The pandemic may signal a return to industrial policy in many countries. Shortages of personal protective equipment (PPE) for essential workers, shortages of medical devices, such as ventilators, together with the often haphazard and inefficient efforts to produce these products during the first wave, may force nations to take a greater interest in their own production and distribution networks. There may be more protectionism, working to ensure that health products deemed essential stay within national borders.

This kind of geopolitical strategy may go beyond healthcare equipment and extend to other types of emergency supplies and preparations, including for war and natural disasters. This will benefit some of the industrial cities that make this kind of equipment, which are often intermediate cities, rather than large urban centres. The French Prime Minister, Jean Castex, announced in July 2020 a new version of national industrial policy, in part based on re-shoring strategic supply chains and in part on sharing subsidies for industries hurt by the pandemic. It remains uncertain whether these and other such efforts will take hold, and the extent to which they will become serious policies as opposed to political signalling at a time when elites are under pressure.

However, the benefits of industrial policy may be limited because of the automation and artificial intelligence necessary to make these new industries competitive and avoid the risk of permanently subsidising them, keeping costs low and requiring fewer human workers. In other words, nations will not see the kind of broad, middle-class and geographically widespread prosperity that accompanied previous waves of strong industrial policy, such as in the aftermath of the Second World War. The inter-regional economic polarisation that has been a fundamental of economic geography since 1980 is likely to continue, or to require much more drastic policies if it is to be tempered.

### Built environment: Changes in urban form, streetscapes, construction/engineering and design

The pandemic is already impacting city centres, principal urban arteries (high streets or main commercial streets) and urban streetscapes. The already-struggling urban retail sector has been dealt a heavy additional blow by lockdowns and the turn to delivery-based consumption, causing countless businesses to contract and fail. High streets may lose their commercial role, instead becoming amenity centres and window-shopping destinations, enticing shoppers to buy online.

Declining demand for offices and co-working spaces may drive down commercial rents and prompt proposals for adaptive reuse, including residential conversion. This process could extend in time, especially if companies shed office space and move to more flexible working systems. These kinds of conversions will depend on financial markets and urban political regimes that are open to these significant, and relatively unprecedented, investments.

The shock from the pandemic has dampened demand and lowered rents for residential apartments in downtowns and central urban neighbourhoods, though the picture is, so far, mixed for real-estate prices. We do not yet know whether the fall in rents is temporary, or how much it will lead to fundamental shifts in the value of real estate as opposed to historically low interest rates and high savings. As noted above, there is likely to be sorting into and out of central cities. Some may accelerate their moves to suburbs or second-rank metros but others, demanding urban buzz, could lead a return to the city, seeking to live close to work and amenities. If mixed work-weeks lead to a net decline in high-income central city employment, however, real-estate and rental prices may fall durably. In this case, artists and creatives could come back to the very neighbourhoods they were forced out of by gentrification.

In any event, the landscape of urban amenities – so important to the formation of urban neighbourhoods, gentrification and urban activity patterns – can be substantially altered, possibly over a period of depressed economic activity and social scarring. Rebuilding the networks of people with all of the ‘new urban skills’ (waiters, mixologists, artisans, entertainers) may be a generation-long process, as individuals currently working in those fields are forced to find other jobs or to await the rebuilding of businesses.

A ‘retail apocalypse’ was already underway prior to the pandemic shock. It has been accelerated by the move to online shopping and this will be permanent. Prior to the pandemic, the notion that streetscape retail would have to become more experiential, or even just something like the frontspace for online shopping, was gaining acceptance. The mix of entertainment-oriented streetscape (restaurants, experiences, performance, galleries and so on) will grow relative to the goods-selling part. A mix of production–living–working spaces could reshape high streets.

Should these changes endure, there will be transformations in future urban planning and building. The regulations for activities on major urban arteries might be modified to allow for more flexible uses of street-fronting space, including housing or live–work spaces. Buildings might be redesigned for health impacts and distancing in shared spaces. New remodelling and building will require vigorous and easy maintenance of high health standards, preparing for future pandemics. The mix of design, urban form, land-use changes and temporary health measures will work itself out over a period of years and suggests a major rethinking of community and urban design ideas that have been dominant in the previous period.

A major concern in the transformation of urban streets and public spaces is whether such ‘new-new urbanism’ will be inclusive and public-oriented, or whether it will be bunkered. Already, responses to climate change in places such as Miami have involved the construction of super-luxury towers that attempt to isolate the rich from the growing impacts of flooding, while diverting future floodwaters onto the less advantaged (Stewart and García-Navarro, 2019). The equity impacts of new ideas about designing-for-resilience should become the subject of a vigorous debate as new regulations and practices are developed (Rodríguez-Pose and Storper, 2020).

## The future of the post-coronavirus city

Many of the potential transformations we describe above will depend on the duration of the pandemic. Already as we write in April 2021, vaccination is occurring rapidly in advanced nations. If the duration is short, cities may return to a new normal that is not very different from the previous normal. Some operational and regulatory changes will persist but the overall structure of urban life will remain mostly unchanged, just as in the Roaring 1920s following the Great Flu, or New York City after 9/11. We may be looking at our own version of the ‘Roaring 2020s’. A century from now, we may think of COVID-19 as yet another ‘forgotten pandemic’, whose impact was horrific in the moment but quickly washed away in the tide of history (Wiseman, 2020).

Making predictions is difficult but, in the analysis above, our primary scenario is that the broad macrogeographical pattern of urbanisation is unlikely to be changed. However, there could be significant intra-metropolitan, neighbourhood-level and daily life changes to cities. This case can be summarised in three main points.

1. There will be long-lasting transformations of work and shopping. The normalisation of remote work will lead to a longer-term shift, with fewer people commuting five days a week at peak hours. Without government intervention, the acceleration of online shopping will threaten the survival of high streets as we know them. And employment opportunities for mid-skill workers will be negatively affected, accelerating the balkanisation of the workforce into highly paid knowledge workers and poorly paid delivery and service workers. Moreover, there may be consequences for urban real estate, including a possible long-term decline in commercial real-estate values. This comes on the heels of the already-unfolding retail apocalypse. The new price gradients may open up a countertrend to commercial and residential gentrification, creating opportunities for new uses that have been excluded from hot spot cities. Streetscapes may be repurposed to promote social distancing.
2. While the pre-eminence of cities will be unaffected, their functions could change. Cities might increasingly become cultural and civic gathering places, rather than shopping destinations or office hubs. More events will take place outdoors, in city streets and plazas, as part of the transformation of city centres into pedestrian and bike havens. Some neighbourhoods losing wealthier residents to the suburbs might gain another character, as young people, artists and creatives take advantage of potentially lower housing costs. However, cities may grow even more unequal than they are today, as both the disease and its economic fallout hit the least advantaged hardest.
3. The winner-take-all economic geography of cities will remain in force. When the pandemic is over, New York and London will still be the world’s great financial centres; the Bay Area its hub of high technology; and Los Angeles its centre for entertainment and film. Shanghai, Tokyo, Singapore, Paris or Toronto will remain alpha or global cities. Even if the downtowns of these cities lose out somewhat to their suburbs and nearby small towns, the general, winner-take-all geography of cities will persist. Most medium-sized cities and rural areas, especially those far from dynamic economic centres, may lose out.

However, two main doubts about the unfolding of the forces above linger. The first is the future of downtowns. As noted, the return to centres in recent decades was triggered by the agglomeration economies of the Third Industrial Revolution. It has been based on a virtuous circle of increased flow of higher-income and young persons into cities combined with the ongoing accumulation of urban amenities. It has had serious collateral effects, such as gentrification and cheek-by-jowl wealth and poverty. This period contrasts dramatically with the decline of American urban centres from the 1960s through the 1980s, where a vicious cycle of white flight, ghettoisation and public housing abandonment, diminishing amenities and deteriorating fiscal capacity led to widespread urban degradation. COVID-19 has hit first and foremost central city areas, with an enormous downward shock to existing amenities. Should this become a more permanent feature of the landscape, it may trigger another self-confirming process of urban abandonment. The short-term response to the pandemic may require decisive urban-focused action just at a time when budgets are strained, in an analogy to the counter-cyclical spending channelled to prevent the pandemic recession developing into a long-term depression. The underlying long-term post-pandemic strengths of central cities we have described are real, but vulnerable right now.

A second overriding concern for both short- and long-term changes to cities is the extent to which they will address structural urban inequalities. We know from experience that past rounds of urban planning and policymaking may have had good intentions for equity and inclusion, but that ‘great planning disasters’ are frequent; ultimately many grand planning schemes merely reproduced structural economic and racial inequalities (Hall, 1982). These inequalities are inscribed in the fundamental workings of urban property and housing markets, and inequalities at work have only been growing in spite of formal efforts at affirmative action and non-discrimination. The interaction of economic, social, racial, urban and territorial inequalities is a powerful nexus. It would be naïve to believe that policies to address the unique conundrums of the pandemic will create greater urban justice without major and specific attention to systemic injustice.

On a more positive note, the pandemic, despite its horrific toll, offers an opportunity to reinvent the way we see the city and, especially, city centres. It offers a window of opportunity where cities can reset and re-energise; where old practices can be called into question. As cities rebuild and recover, they will be forced to rethink their functions and how they can best be fulfilled. At the same time, they can pilot efforts to confront the widening chasms between classes and neighbourhoods and prepare for the many threats of climate change (Zipper, 2020).

The earliest signs of reinvention come from European cities carving out street space for bicycles, scooters and pedestrians (McAuley and Spolar, 2020). In California, activists and politicians are exploring new long-term strategies for housing the homeless, potentially purchasing hotels already used to thin the population of homeless shelters. These are just the beginning of many more such efforts to reinvent cities. As the pandemic continues, the creativity and innovation of humankind will remain most prominently displayed in big cities (Levin, 2020).

All of the forces described – behavioural, economic, engineering, design, societal – will play out and interact. We remain in a period of extended social experimentation, with households, business, the professions and the public sector all in the game. Cities have become laboratories in new ways to govern and shape their futures. There may be considerable variety in the responses, according to differences in geography, wealth, and political and social preferences. This experimental period is likely to endure post-pandemic and it will be some time before anything like best practices for the post-COVID city are identifiable. In this new open and experimental period, we must keep an eye on the fundamental line of division between unequal and bunkered urbanism and a new urbanism aiming for inclusion and economic and social revival in a more just way; a truly resilient new post-COVID city.
